# STATAWAARS: a promoter motif associated with spatial expression in the major effector-producing tissues of the plant-parasitic nematode *Bursaphelenchus xylophilus*

**DOI:** 10.1186/s12864-018-4908-2

**Published:** 2018-07-27

**Authors:** Margarida Espada, Sebastian Eves-van den Akker, Tom Maier, Vijayapalani Paramasivan, Thomas Baum, Manuel Mota, John T. Jones

**Affiliations:** 1 NemaLab, ICAAM – Instituto de Ciências Agrárias e Ambientais Mediterrânicas, Universidade de Évora, Instituto de Investigação e Formação Avançada, Universidade de Évora, Pólo da Mitra, Ap. 94, 7006-554 Évora, Portugal; 2 Cell and Molecular Sciences Group, The James Hutton Institute, Invergowrie, Dundee, DD2 5DA UK; 3 Department of Plant Sciences, University of Cambridge, Cambridge, CB3 1AB UK; 4 Department of Plant Pathology and Microbiology, Iowa State University, Ames, 50011 USA; 5 NemaLab, ICAAM - Departamento de Biologia, Instituto de Ciências Agrárias e Ambientais Mediterrânicas, Universidade de Évora, Escola de Ciências e Tecnologia, Universidade de Évora, Pólo da Mitra, Ap. 94, 7006-554 Évora, Portugal; 6 School of Biology, University of St Andrews, North Haugh, St Andrews, KY16 9TZ UK

**Keywords:** *Bursaphelenchus xylophilus*, Effectors, Gene regulation, Promoter motif, Pharyngeal gland cells, Host-pathogen interaction, Plant-parasitic nematode

## Abstract

**Background:**

Plant-parasitic nematodes cause severe damage to a wide range of crop and forest species worldwide. The migratory endoparasitic nematode, *Bursaphelenchus xylophilus*, (pinewood nematode) is a quarantine pathogen that infects pine trees and has a hugely detrimental economic impact on the forestry industry. Under certain environmental conditions large areas of infected trees can be destroyed, leading to damage on an ecological scale. The interactions of *B. xylophilus* with plants are mediated by secreted effector proteins produced in the pharyngeal gland cells. Identification of effectors is important to understand mechanisms of parasitism and to develop new control measures for the pathogens.

**Results:**

Using an approach pioneered in cyst nematodes, we have analysed the promoter regions of a small panel of previously validated pharyngeal gland cell effectors from *B. xylophilus* to identify an associated putative regulatory promoter motif: STATAWAARS. The presence of STATAWAARS in the promoter region of an uncharacterized gene is a predictor that the corresponding gene encodes a putatively secreted protein, consistent with effector function. Furthermore, we are able to experimentally validate that a subset of STATAWAARS-containing genes are specifically expressed in the pharyngeal glands. Finally, we independently validate the association of STATAWAARS with tissue-specific expression by directly sequencing the mRNA of pharyngeal gland cells. We combine a series of criteria, including STATAWAARS predictions and abundance in the gland cell transcriptome, to generate a comprehensive effector repertoire for *B. xylophilus*. The genes highlighted by this approach include many previously described effectors and a series of novel “pioneer” effectors.

**Conclusions:**

We provide a major scientific advance in the area of effector regulation. We identify a novel promoter motif (STATAWAARS) associated with expression in the pharyngeal gland cells. Our data, coupled with those from previous studies, suggest that lineage-specific promoter motifs are a theme of effector regulation in the phylum Nematoda.

**Electronic supplementary material:**

The online version of this article (10.1186/s12864-018-4908-2) contains supplementary material, which is available to authorized users.

## Background

Plant-parasitic nematodes (PPN) infect a broad range of plants of agricultural and economic importance. They display a wide range of interactions with their hosts and many are biotrophic pathogens. The pinewood nematode, *Bursaphelenchus xylophilus*, is a migratory endoparasitic nematode that causes extensive damage to forestry across many parts of the world. The life cycle of this nematode is complex and includes fungal- and plant-feeding stages, as well as a stage that is vectored to new hosts by an insect, most often the longhorn beetle *Monochamus* spp. (reviewed by [1, 2]). The fungal-feeding stage of the nematode feeds on endophytic fungi present in dead or dying pine trees. As food availability declines, the nematode enters a survival stage which locates pupae of *Monochamus* and settles within the tracheae or beneath the elytra of the adult beetle as it emerges from the pupal chamber. The beetle may migrate to another tree colonized by fungi or may feed on living trees. In the latter case the nematode leaves the beetle and infects the host tree, feeding on parenchymal and epithelial cells. Nematodes migrate, feed, and reproduce within the host causing extensive damage both directly, due to their feeding activities, and indirectly as a result of disruption of water transport due to cavitation of infected tissues. Under appropriate environmental conditions, most notably in hot climates, death of infected trees can occur within weeks of infection [1, 3].

Like other plant pathogens, the interactions of PPN with their host plants are mediated by effectors, secreted proteins originating from pharyngeal gland cells that are secreted into the host through the stylet [4, 5]. These proteins enable the nematode to successfully feed, reproduce and migrate inside the host. Advances in genomics and transcriptomics have allowed insights into the types of effectors required for parasitism by *B. xylophilus*. A range of plant cell-wall degrading enzymes and modifying proteins, which presumably facilitate invasion and migration, have been identified including cellulases [6], pectate lyases [7] and expansins [8]. More recently, RNAseq analysis of nematodes after infection of trees revealed that a range of antioxidant and detoxification proteins are deployed as effectors during infection [9]. This analysis also identified a number of pioneer effector sequences that have no similarity to other previously identified sequences but that encode secreted proteins which are specifically expressed in the gland cells of the nematode. The importance of effectors in the life cycle of PPN has led to a range of approaches being used for their identification. Perhaps the most efficacious of the methods used to date has been direct analysis of the genes expressed in the pharyngeal gland cells. Initially this was achieved through Expressed Sequence Tag (EST) analysis of cloned cDNA made from RNA extracted from these tissues (e.g. [10, 11]). A method was subsequently developed for micro-aspiration of gland cell contents followed by RNAseq analysis and has been used to identify effectors from a range of PPN [12].

Genes encoding PPN effectors are primarily, and specifically, expressed in the pharyngeal gland cells (e.g. [4]). This tissue-specific gene expression implies the existence of a shared regulatory mechanism. In *Caenorhabditis elegans* and *C. briggsae*, various non-coding promoter motifs have been shown to describe tissue-specific expression patterns (for example muscle) [13]. More recently, this approach was applied to plant-parasitic nematodes: the Dorsal Gland box (DOG box) is a 6 bp promoter motif that is associated with, and can be used to predict, genes specifically expressed in the dorsal gland cell of the potato cyst nematodes *Globodera rostochiensis* and *G. pallida* [14]. Analysis of promoter motifs offers a powerful tool for identification of novel effectors a priori [15].

In spite of the progress described above, our understanding of the effectors produced by *B. xylophilus* and the mechanisms by which it infects its hosts is incomplete. The greatest progress in terms of identification and functional characterisation of effectors has been made with the sedentary endoparasitic cyst forming (*Heterodera* and *Globodera* spp.) and root-knot nematodes (*Meloidogyne* spp.). *Bursaphelenchus xylophilus* is distantly related to both these groups, has an independent origin of plant-parasitism, and has a very different mode of parasitism. Taken together, this would suggest neither extensive overlap in effector repertoires, nor “conserved” regulatory mechanisms: recent studies support both these suggestions [9, 14, 16].

Here we have identified a novel promoter motif associated with genes expressed in the pharyngeal gland cells of *B. xylophilus,* and used this motif to identify candidate effectors from the genome. We directly sequenced the transcriptome of the pharyngeal gland cells, to validate and refine motif-based predictions, and constructed a comprehensive superset of effectors for this species.

## Results

### Identification of a promoter motif associated with pharyngeal gland cell expression

Recent analysis of the genome sequence of *G. rostochiensis* allowed identification of a non-coding promoter motif (the DOG box, ATGCCA) associated with genes expressed in the dorsal pharyngeal gland cell [14] which has subsequently been used as a tool to predict effectors in this genus. We sought to determine whether a similar approach could be used to identify a motif associated with genes expressed in the pharyngeal gland cells of *B. xylophilus* which, although it is also a plant-parasite, is distantly related to *G. rostochiensis* and is located in a different phylogenetic clade in the Nematoda [17]*.* To identify potential regulatory elements associated with genes expressed in the gland cells, we assembled a training set of 42 genes (Additional file 1: Table S1) for which gland cell expression had been previously validated in a range of studies [6, 7, 9, 18, 19]. These sequences included plant cell wall-degrading enzymes as well as novel effectors identified in our previous work [9].

**Fig. 1:**
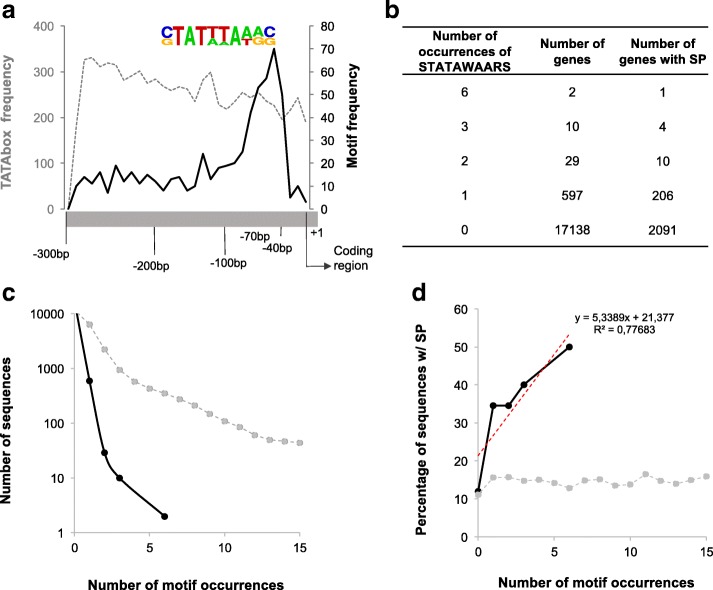
Features of STATAWAARS and associated genes in the *B. xylophilus* genome. **a** Sequence logo of the (original) motif STATWWAARS (in colour) showing the consensus and variable sites. The motif (black line) peaks around 70 bp upstream of the predicted coding region while the TATA box has a broad distribution (grey dashed line); **b**: Number of genes that have the presence of the (refined) motif STATAWAARS and the number of genes that have the presence of signal peptide (Fisher’s exact test *p*-value of 1e-05; significant for *p* < 0.01) (**c**) Number of sequences with various numbers of iterations of STATAWAARS (black line) as compared to the TATA box (grey line), and (**d**) proportion of genes with STATAWAARS or TATA box that are also predicted to encode a signal peptide for secretion. A trend line predicts the increasing probability of the presence of signal peptide in the sequences that have the presence of the motif

The 300 bp promoter region of each of the 42 effectors in the training set was extracted from the genome and compared to the promoter regions of the 17,735 other predicted genes in the genome of *B. xylophilus*. Employing a differential motif discovery algorithm, we identified a promoter motif that was highly enriched in the effector set (Fisher’s exact test; *p*-value: 1e-18). This motif was present in 62% of effector promoters, with an average of one motif per promoter. This motif, which has the consensus sequence STATWWAWRS, has six variable loci indicated by the DNA ambiguity code ([C|G]TAT[T|A][T|A]A[T|A][G|A][C|G]), meaning that a number of variants of this sequence are potentially present in the *B. xylophilus* genome (Fig. 1a; Table 1). We analyzed each of the variants individually but found no patterns of association with specific gene classes (not shown). One such variant at position 5 (T) showed no preferential association with effectors, and position 8 is invariably adenine (A) in all effectors (Table 1). Therefore, a refined motif, STATAWAARS, was used for all further analyses (Table 1).

**Table 1:**
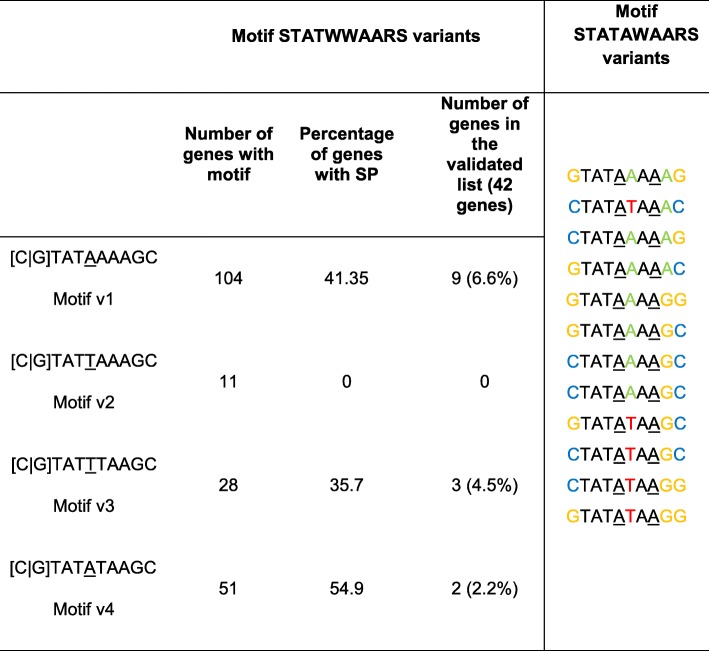
Analysis of the motif STATWWAWRS variants (v) in position 5, nucleotides T or A (underlined; left column). The presence of a T is not associated with any sequence with the presence of signal peptide. Different combinations of the variants from motif STATAWAARS (right column). In both locus 5 and locus 8 adenine (underlined) is the preferential allele

### STATAWAARS as a predictor of secretory proteins and gland cell expression

To determine the efficacy of using STATAWAARS to predict effectors on a genome-wide scale, we extracted the 300 bp immediately 5′ of all predicted coding regions in the genome (termed the promoter region). These promoter regions were analysed for the presence of STATAWAARS and the TATA box (TATAAA) as a sequence-similar control.

The STATAWAARS motif was present at least once in the 300 bp upstream of the predicted coding region of 597 genes (from a total set of 17,735 predicted *B. xylophilus* genes). Most STATAWAARS-containing promoters have only a single motif (*n* = 556), while two promoters contained the maximum of six elements (Fig. 1b). The TATAAA motif was present in the promotor regions of 6417 of the 17,735 *B. xylophilus* predicted genes with up to 15 iterations per promoter region (Fig. 1c). Occurrences of STATAWAARS peaked between 40 to 70 bp upstream of the coding region which is different when compared to the position of the TATA box in relation to the start codon (Fig. 1a). The most abundantly represented classes of genes that had the STATAWAARS-motif are peptidases (cysteine and aspartic) and genes that have no similarity to others in databases, i.e. pioneer sequences (Additional file 2: Table S2).

Genes containing a STATAWAARS motif in their promoter region are more likely to also encode a protein with a predicted signal peptide for secretion, a canonical feature of plant-parasitic nematode effectors. Thirty four percent of the 597 STATAWAARS-containing genes (Additional file 2: Table S2) encode a protein with a predicted signal peptide (*n* = 206), compared to just 15.6% of those associated with the TATAAA motif, and 12.7% of all known genes in the *B. xylophilus* genome [9] (Fig. 1d). To provide an estimate on the likelihood of the apparent enrichment, the chance of randomly selecting 597 *B. xylophilus* genes where 34% encode a signal peptide for secretion was empirically derived using an iterative approach. We selected 597 *B. xylophilus* genes at random, one million times. The iteration with the highest proportion of proteins with a signal peptide was less than 34% (21.7%), suggesting a probability of < 1 in a million, or *p* < 0.000001. In addition, the more copies of STATAWAARS in the promoter region, the greater the percentage of associated genes that encode a signal peptide for secretion (Fig. 1b, d). Proteins with a signal peptide are therefore over-represented (Fisher’s exact test *p*-value< 0.01) in the sequences that are downstream of the STATAWAARS motif, as would be expected if this motif is associated with genes expressed in the effector-producing secretory gland cells.

**Fig. 2:**
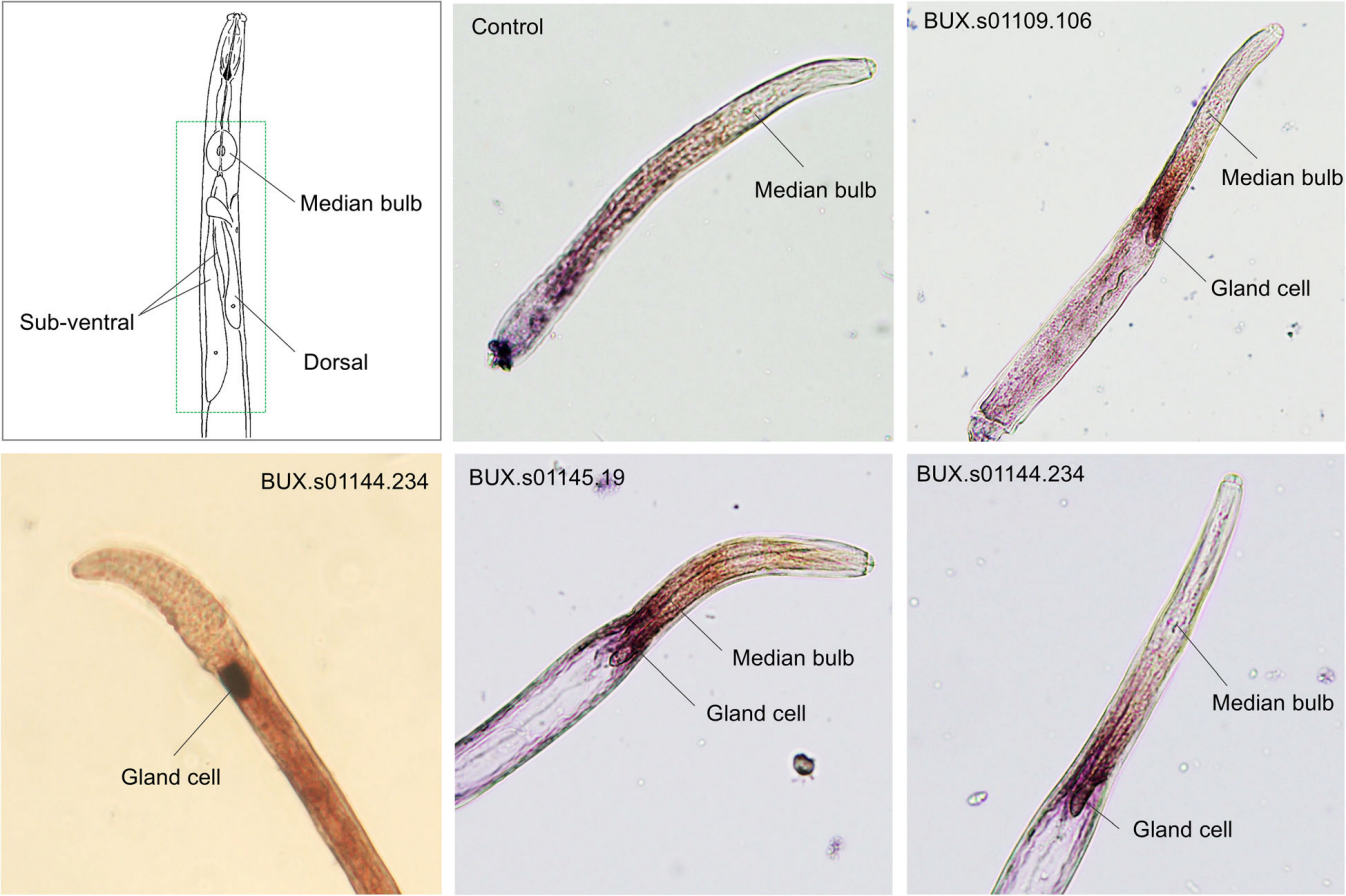
Localisation of candidate effector genes in the pharyngeal gland cells by in situ hybridisation. Each of these genes is associated with the STATAWAARS motif. Sequence similarity analysis with BlastP showed that BUX.s01144.234 is similar to a thaumatin-like protein, BUX.s01109.106 and BUX.s01147.71 are similar to transthyretin-like proteins and BUX.s01145.19 is similar to a lipase found in *C. briggsae* (CBR-LIPL-1). *B. xylophilus* pharyngeal gland cells illustration adapted from [36] (top left). Nematodes were hybridised with an anti- sense and sense (control) DIG-labeled cDNA probes

In order to determine whether the STATAWAARS motif can act as a predictor of sequences expressed in the gland cells of *B. xylophilus,* we used in situ hybridisation  to examine the spatial expression pattern of novel STATAWAARS-containing genes (i.e. those that had not previously been studied) in mixed-stage nematodes. For this analysis we selected ten genes that were abundantly expressed in the nematode (as assessed on the basis of previous RNAseq data [9]). Using this approach, we experimentally validated the ability of the STATAWAARS motif to act as a predictor of gland cell expression for seven out of these ten *B. xylophilus* genes including BUX.s01144.234, a sequence similar to a thaumatin-like protein; BUX.s01109.106 and BUX.s01147.71, sequences similar to a transthyretin-like protein; and BUX.s01145.19, a sequence similar to a lipase found in *C. briggsae* (CBR-LIPL-1, Fig. 2). While some (but not all) transthyretin-like proteins have been described as expressed in the gland cells of other plant-parasitic nematodes [20], there are no previous reports of thaumatins or lipases being associated with the gland cells of nematodes. Taken together, these data suggest that the STATAWAARS promoter motif is a useful additional criterion to facilitate effector prediction in *B. xylophilus*.

**Fig. 3:**
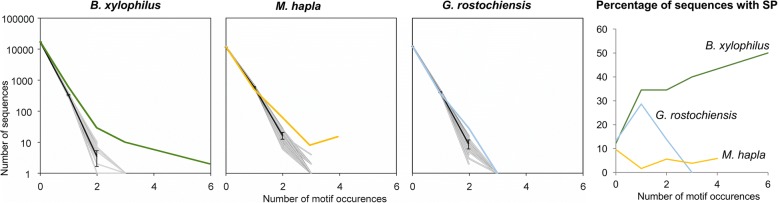
The STATAWAARS motif in *B. xylophilus* and two sedentary plant-parasitic nematodes, *Meloidogyne hapla* and *Globodera rostochiensis*. The motif is present in the genomes of all three species. The Frequency of motif occurrence in *B. xylophilus* (Green) appears to differ from an analysis of 1000 shuffled promoter sequences (Grey). The Frequency of motif occurrence in *M. hapla* (yellow) differs somewhat from an analysis of 1000 shuffled promoter sequences (Grey). The Frequency of motif occurrence in *G. rostochiensis* (Blue) is largely similar to an analysis of 1000 shuffled promoter sequences (Grey). The grey lines represent 250 shuffles and the black line represents the average of the shuffles (error bars indicate standard deviation (if the average is greater than 1)). Although the motif is present in the promoter regions of *G. rostochiensis* (blue line) and *M. hapla* (yellow line) multiple copies of the motif are not associated with the presence of a signal peptide (SP). For *B. xylophilus* (Green), the more motifs present in the promoter region, the more likely the corresponding gene encodes a signal peptide

To determine whether the STATAWAARS motif can also be used to predict effectors from other plant-parasitic nematodes of independent evolutionary origin, we analyzed the genomes of a cyst nematode *G. rostochiensis* and the root-knot nematode *Meloidogyne hapla*. In each species, we compared the number of canonical STATAWAARS motifs in the promoter regions of all genes with the number of canonical STATAWAARS motifs that occur at random (by randomizing all promoter regions 1000 times). In *B. xylophilus*, multiple copies of STATAWAARS in the promoter region occur more frequently than random, and the more copies in the promoter region, the more likely it is that the corresponding gene encodes a signal peptide for secretion (Fig. 3). The promoter regions of some *G. rostochiensis* genes do contain the STATAWAARS motif, but this frequency is no higher than expected by chance and there is no consistent association with the number of STATAWAARS motifs and signal peptide prediction. Finally, while the promoter regions of *M. hapla* genes do appear to encode more STATAWAARS motifs than expected by chance, there is also no consistent association with the signal peptide predications (Fig. 3). Taken together with published data, this suggests that the DOG box is apparently specifically associated with dorsal gland expression in cyst nematode genes but is not in those of pinewood or root-knot nematodes [14], and that the STATAWAARS motif identified here specifically associated with dorsal gland expression in pinewood nematode genes but not in those of cyst or root-knot nematodes.

### Analysis of the transcriptome of purified *B. xylophilus* pharyngeal gland cells

To further validate the association of STATAWAARS with gland cell expression, and expand the effector repertoire of *B. xylophilus*, we dissected gland cells from mixed stage cultured nematodes and directly sequenced their mRNA contents as previously described [12]. From a single library preparation, two sequencing runs were carried out (BX-1 and BX-2), resulting in 124 and 143 million reads respectively. In each case approximately 30% of the reads mapped to the *B. xylophilus* genome (Additional file 3: Table S3). This relatively low mapping rate is probably related to the amplification required to generate sufficient material for sequencing with the majority of the unmapped reads derived from RNA used for removal of rRNA from the sample (not shown). Nevertheless, the remaining 35–40 million reads in each sequencing run represent sufficient coverage of the exome of the pharyngeal glands for further analysis.

**Fig. 4:**
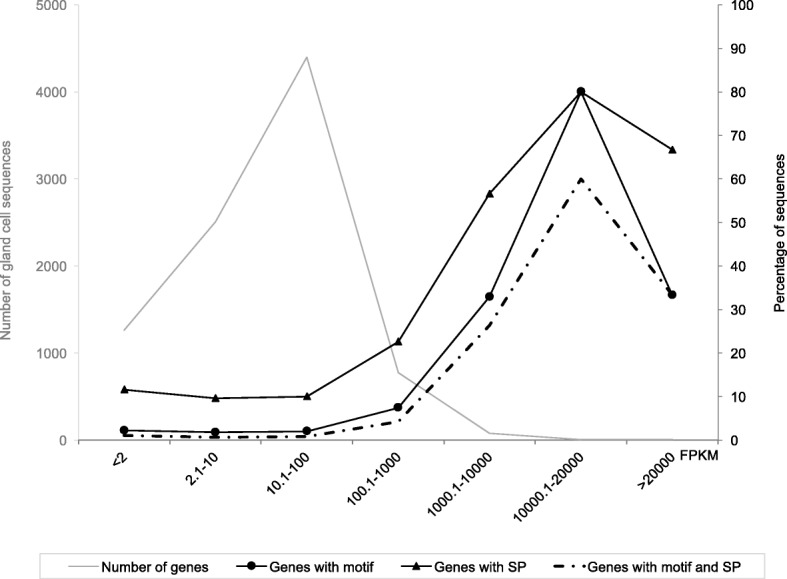
Distribution of representation in the gland cell transcriptome and percentage of those genes that have a signal peptide (SP) or STATAWAARS motif. Number of the genes represented in the gland cell transcriptome data set (grey line) and the percentage of proteins with a signal peptide (black lines). The representation in the gland cell library is divided into bins according to the FPKM value (Fragments per kilobase million, X axis). The presence of signal peptide and the motif STATAWAARS (dashed black line) is increased in the most highly represented bins

Due to the technical difficulties in extracting gland cells from nematode homogenate, it is important to note that this library neither exclusively contains transcripts from the gland cells (mRNA from other tissues will be present), nor do all reads originate from transcripts that are exclusively expressed in the gland cells (probably most genes expressed in the gland cells will also be expressed in other tissues, e.g. housekeeping functions). We reason that the abundantly represented transcripts are thus likely expressed in the gland cells. Consistent with this, the majority of genes in the *B. xylophilus* genome were represented by at least one read in the gland cell transcriptome library. The number of reads mapped per gene was used to categorize genes based on how abundantly they were represented in the library (< 2, 2.1–10, 10.1–100, 100.1–1000, 1000.0–10,000 and > 20,000 FPKM, fragments per kilobase million). The number of genes in each category increases with representation up to 100 FPKM (*n* = 4399), and then sharply decreases in all other subsequent categories (Fig. 4). Strikingly, the proportion of the sequences that have a predicted signal peptide is strongly positively correlated with representation, peaking at 10000 FPKM: in the three most highly represented categories, the proportion of putatively secreted proteins was much higher than that of the remaining *B. xylophilus* genes (Fig. 4). Moreover, the proportion of genes that contain STATAWAARS in their promoter is also strongly positively correlated with increased representation, and similarly peaks at 10000 FPKM (Fig. 4). Finally, more than half of the genes which encode STATAWAARS in their promoter are represented in the gland cell transcriptome. Taken together, these data further validate the association of STATAWAARS with expression in the gland cells.

Given that a signal peptide is a canonical feature of effectors, it stands to reason that those genes most highly represented in the gland cell transcriptome library and that encode both a signal peptide for secretion and contain the STATAWAARS motif are indeed effectors. As would be expected, increased abundance in this library is associated with an increase in features associated with effectors (signal peptide and STATAWAARS).

To determine whether other promoter motifs are also associated with gland cell expression in *B. xylophilus,* the 300 bp promoter regions of the 30 most abundant genes in the gland cell transcriptome were extracted (Additional file 4: Table S4) and analysed for overrepresented motifs. Encouragingly, STATAWAARS was the most highly represented motif (HOMER motif discovery algorithm; *p*-value of 1e-11) in this new input dataset and was present in the promoter region of 40% of the genes. No other novel motifs were revealed as a result of this analysis. Since there is limited overlap between this gene set and the original training set used to identify the STATAWAARS motif, this provides a strong independent validation for the association of the motif with genes expressed in the gland cells (Additional file 5: Table S5).

### Candidate effectors of *B. xylophilus*

**Fig. 5:**
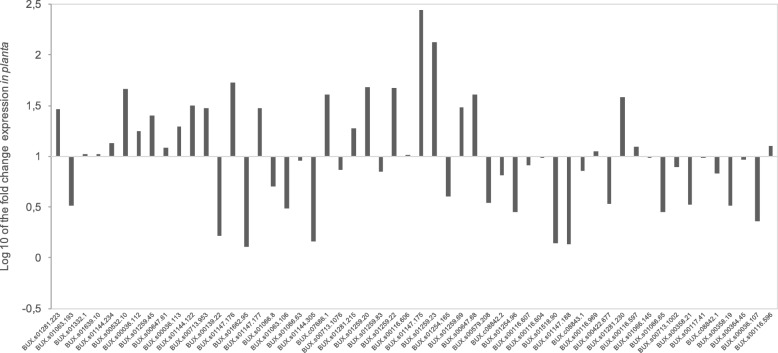
Comparison between the transcriptomic profile (log 10 of the fold change of the expression upon infection of the plant host) and the 54 most abundant genes with signal peptide and presence of the STATAWAARS motif. Twenty-five genes are over the threshold line representing 46% of these genes

The transcriptome data and list of genes associated with the STATAWAARS promoter motif were subsequently used to generate a comprehensive repertoire of putative *B. xylophilus* effectors using the following criteria: represented in the gland cell transcriptome dataset (FPKM> 100), encode a protein with a predicted signal peptide at the N-terminus, and have at least one occurrence of the STATAWAARS motif in the genomic region 1000 bp upstream of the predicted start codon. A total of 54 sequences fulfilled these criteria (Table 2). A comparison with a previous transcriptome analysis of this nematode [9] shows that almost half of these sequences are upregulated *in planta* (Fig. 5), consistent with a role in parasitism. In addition, approximately 52% of these putative effector sequences were identified in a proteomic analysis of secreted proteins of *B. xylophilus* [21], a considerable enrichment compared to the 8.4% of all *B. xylophilus* proteins that were identified in this analysis, and independent validation of the approach.

**Table 2:** Candidate effectors from *B. xylophilus*: 54 genes are represented in the gland cell transcriptome (FPKM > 100) and have both a signal peptide and at least one occurrence of the STATAWAARS motif in the promoter region. The predicted protein domains were determined by BlastP (threshold of 1 e-04)

GeneID	Up-regulated *in planta*	Expression *in planta*	Expression in gland cells	Expression in gland cells confirmed	Predicted protein function
BUX.s01281.223	+	103.9	20,907.19	+	gi|171854685|dbj|BAG16532.1| expansin-like protein [*B. xylophilus*]
BUX.s01063.193	–	1934.67	14,530.98		gi|510850849|gb|EPB67435.1| Transthyretin-like family protein [*Ancylostoma ceylanicum*]
BUX.s01332.1	+	55.73	12,613.75		Not known
BUX.s01639.10	+	55.73	12,613.75	+	Not known
BUX.s01144.234	+	171.06	8343.09	+	gi|762079885|ref.|XP_011414138.1| PREDICTED: thaumatin-like protein 1b [*Crassostrea gigas]*
BUX.s00532.10	+	128.23	7085.05		gi|507051878|ref.|WP_016122867.1| LPXTG-domain-containing protein cell wall anchor domain [*Bacillus cereus*]
BUX.s00036.112	+	393.48	6830.64	+	gi|50872001|dbj|BAD34545.1| beta-1,4-endoglucanase [*B. xylophilus*]
BUX.s01259.45	+	84.44	6760.45	+	gi|657202143|gb|AID50178.1| cysteine protease family cathepsin 1 [*B. mucronatus*]
BUX.s00647.61	+	297.20	5542.85	+	Not known
BUX.s00036.113	+	322.72	4504.83	+	gi|50871999|dbj|BAD34544.1| beta-1,4-endoglucanase [*B. xylophilus*]
BUX.s01144.122	+	127.83	4148.47	+	Not known
BUX.s00713.953	+	176.45	3997.31		Not known
BUX.s00139.22	–	2224.90	3881.41	+	Not known
BUX.s01147.176	+	85.58	3291.83		gi|657202143|gb|AID50178.1| cysteine protease family cathepsin 1 [*B. mucronatus*]
BUX.s01662.95	–	251.02	2564.99		gi|541044673|gb|ERG83573.1| vitellogenin-6 [Ascaris suum]
BUX.s01147.177	+	119.82	2190.46	+	gi|657202143|gb|AID50178.1| cysteine protease family cathepsin 1 [*B. mucronatus*]
BUX.s01066.8	+	987.43	2107.94		gi|308493871|ref.|XP_003109125.1| CRE-LYS-8.1 protein [*C. remanei*]
BUX.s01063.106	–	45.41	1816.8	+	Not known
BUX.s01066.63	+	138.92	1437.37	+	gi|68226394|dbj|BAE02683.1| beta-1,3-endoglucanase [*B. xylophilus*]
BUX.s01144.305	–	25.97	1383.88		Not known
BUX.c07686.1	+	5.18	1345.1		Not known
BUX.s00713.1076	–	30.03	1270.5		gi|657202143|gb|AID50178.1| cysteine protease family cathepsin 1 [*B. mucronatus*]
BUX.s01281.215	+	52.28	1076.62		gi|171854689|dbj|BAG16534.1| expansin-like protein [*B. mucronatus*]
BUX.s01259.20	+	209.06	936.56	+	gi|82175173|dbj|BAE48370.1| pectate lyase [*B. xylophilus*]
BUX.s01259.83	–	21.80	785.02	+	gi|657202143|gb|AID50178.1| cysteine protease family cathepsin 1 [*B. mucronatus*]
BUX.s01259.22	–	6.95	708.17		gi|82175173|dbj|BAE48370.1| pectate lyase [*B. xylophilus*]
BUX.s00116.606	+	33.60	677.13	+	gi|402314083|gb|AFQ55440.1| venom allergen-like protein [*Ditylenchus destructor*]
BUX.s01147.175	+	17.44	664.85		gi|657202143|gb|AID50178.1| cysteine protease family cathepsin 1 [*B. mucronatus*]
BUX.s01259.23	+	34.02	649.9		gi|82175173|dbj|BAE48370.1| pectate lyase [*B. xylophilus*]
BUX.s01254.165	–	122.36	606.76		Not known
BUX.s01259.69	+	7.37	599.06		Not known
BUX.s00647.68	+	50.73	586.73		Not known
BUX.s00579.208	–	59.63	548.56		Not known
BUX.c08842.2	–	3.06	497.53		Not known
BUX.s01254.96	–	414.47	480.46		Not known
BUX.s00116.607	–	26.29	460.6		gi|657202143|gb|AID50178.1| cysteine protease family cathepsin 1 [*B. mucronatus*]
BUX.s00116.604	+	4.32	396.78		gi|657202143|gb|AID50178.1| cysteine protease family cathepsin 1 [*B. mucronatus*]
BUX.s01518.90	–	0.95	395.68		Not known
BUX.s01147.188	–	100.22	380.57	+	gi|802707556|gb|KKA71696.1| vit-6, partial [*Pristionchus pacificus*]
BUX.c08843.1		2.99	344.18		Not known
BUX.s00116.969	+	21.14	297.48	+	Not known
BUX.s00422.677	–	2685.78	289.34		Not known
BUX.s01281.230	+	98.77	270.09	+	gi|674842627|gb|AIL31417.1| expansin-like protein [*B. xylophilus*]
BUX.s00116.597	+	1182.49	263.89		gi|685827799|emb|CEF62721.1| Hypothetical protein SRAE_1000099100 [*Strongyloides ratti]*
BUX.s01066.145	+	68.54	201.78	+	gi|68226394|dbj|BAE02683.1| beta-1,3-endoglucanase [*B. xylophilus*]
BUX.s01066.65	–	0.09	185.69		gi|68226394|dbj|BAE02683.1| beta-1,3-endoglucanase [*B. xylophilus*]
BUX.s00713.1002	+	28.14	168.39	+	gi|541044223|gb|ERG83158.1| gut esterase 1 [*Ascaris suum*]
BUX.s00358.21	–	536.25	152.96		Not known
BUX.s00117.41	+	35.10	140.6		Not known
BUX.c08842.1	+	13.08	123.5		gi|657202143|gb|AID50178.1| cysteine protease family cathepsin 1 [*B. mucronatus*]
BUX.s00358.19	–	551.47	120.75	+	Not known
BUX.s00364.45	+	13.86	118.38		Not known
BUX.s00036.107	–	0.52	117.17		Not known
BUX.s00116.596	+	199.43	115.16		Not known

The *B. xylophilus* effector list includes many previously verified effectors, including several cellulases, beta-1,3-endoglucanases, pectate lyases, expansins (one of which is the most abundant sequence in the gland cell transcriptome dataset), venom allergen proteins and several pioneer sequences for which gland cell expression was subsequently experimentally verified (Figs. 2 and 4). Forty-two of the sequences on this list have been analysed by in situ hybridisation to date; 22 of these are expressed in the gland cells with the majority of the other sequences tested showing no detectable signal. This provides a level of reassurance that the effector list reflects the biology of *B. xylophilus* and that these as yet uninvestigated sequences merit further study. In addition, several proteinases and three transthyretin-like proteins (including the second most abundant sequence in the gland cell transcriptome) are present on the list. More than half of the sequences on the effector list are pioneers, which have no similarity to other sequences in various publically accessible databases. This reflects similar studies on other plant-parasitic nematodes which have shown that a large proportion of effectors are novel sequences (reviewed by [22]).

This list is unlikely to include all effectors, as some genuine effectors may not have been expressed at the time we sampled gland cell material and/or may be under the control of other as yet undetected gland cell promoters. However, this study provides a major scientific advance in the area of effector regulation and a comprehensive and robust list of candidate effectors from *B. xylophilus* for future studies.

## Discussion

Effector identification is an important component in understanding the mechanisms underlying infection of plants by pathogens, including PPN. In the last decades, there have been considerable efforts made in finding new approaches to identify effectors and further understand their functional role in the disease process. For PPN these include direct sequencing of gland cell RNA and analysis of promoter elements. In this study, we have combined these two approaches to identify a more comprehensive effector list from *B. xylophilus.* Using a validated pharyngeal gland cell effector subset, we identified a putative regulatory promoter motif (STATAWAARS) that is associated with expression in the gland cells. This sequence was distinct from the DOG box previously identified in *Globodera* species [14]. While STATAWAARS is present in the genomes of all three PPNs that we examined, the motif is only consistently associated with putatively secreted proteins in *B. xylophilus,* suggesting independent mechanisms of effector regulation in these species. Similar comparative studies in other nematode species may be informative. However, there are at present no published genome sequences for species that are closely related to *B. xylophilus,* such as *B. mucronatus or B. cocophilus*. Although genomes of other migratory endoparasitic nematodes have been sequenced, the assemblies for these are not publically available. In addition, although these nematodes share some features of parasitism with *B. xylophilus* they are distantly related, have an independent origin of parasitism, and are unlikely have similar mechanisms of effector regulation.

Following the identification of the STATAWAARS motif, we were able to confirm that previously uncharacterized genes associated with the motif were expressed in the gland cells. This confirms that, as for the DOG box of *G. rostochiensis,* the STATAWAARS promoter motif can be used to predict new candidate effectors from *B. xylophilus*. The DOG box was apparently specific to one of the two sets of gland cells of *Globodera* cyst nematodes, the dorsal gland. By contrast, STATAWAARS appears to be associated with effectors produced in both sets of pharyngeal gland cells of *B. xylophilus*. Sequencing of libraries made from separated subventral and dorsal glands would be required to investigate the presence of gland cell-specific promoter elements in *B. xylophilus*.

A similar approach, based on identification of promoters associated with genes expressed in specific tissues and/or at specific life cycle stages, may be of benefit if applied to other pathogens for which identifying effectors is difficult. For example, although it is relatively straightforward to identify effectors from oomycetes (based on the presence of an RxLR motif associated with a signal peptide) [23] and bacterial plant pathogens (based on the presence of a type 3 signal sequence) [24], identifying effectors from fungal plant pathogens and aphids is considerably more difficult as, like nematodes, no known protein motif is associated with effectors from these organisms. Given a sufficiently robust training set of known effectors it may be feasible to identify novel effectors in these systems using a similar approach. This is particularly pertinent for aphids, as they have conceptually similar effector-producing tissues, the salivary gland cells [25].

We also applied a complementary approach to identify *B. xylophilus* effectors by sequencing mRNA extracted directly from dissected gland cells. This approach has been used successfully with other PPN [12]. The main benefit of this approach is the ability to directly analyse gland cell tissues, giving a high probability of identifying genuine effectors. Here we find that the ability of this approach has a relatively discrete signal to noise ratio, above which effectors and effector-like sequences are readily identified. Given the technical difficulties of this approach, we predict the precise location of this discrete boundary will vary between gland cell sequencing experiments and should be empirically derived for each new gland cell sequencing experiment in order to avoid false positives. Given that known effectors are among the most abundant sequences represented, this provides a degree of confidence that other abundantly represented secreted proteins of unknown function merit further investigation. Several such sequences were subsequently validated as being expressed in the gland cells, a hallmark of nematode effectors, by in situ hybridisation. In contrast, it is clear that this approach: 1) may not identify all effectors; and 2) be may be contaminated with other nematode body parts due to the technically challenging experimental procedure involved. Both of these factors, coupled with the fact that we were unable to sample parasitic stage nematodes extracted from trees, suggest that some false negatives and positives are unavoidable. The combination of criteria used for effector prediction herein (STATAWAARS, representation in the gland cell transcriptome, signal peptide) mitigate against these risks.

Given the potential drawbacks of each approach used in isolation, we aimed to identify a comprehensive list of effectors by bringing together the gland cell transcriptome and promoter data to cross validate one another. The final list of effectors consisted of 54 sequences that are represented in the gland cell transcriptome with the motif in the region upstream of the coding sequence and which had a predicted signal peptide. Many of the sequences (approx. 35%) on this list are pioneer sequences that have no sequence similarity to others characterized in the databases. This is in keeping with studies on other PPN which have shown that a large proportion of effectors are novel sequences. For example, 38 of 53 confirmed effectors of *H. glycines* and 28 of 37 effectors from *M. incognita* identified in the first studies of these nematodes were pioneers [10, 11]. Similarly, analysis of *G. pallida* [26] and *G. rostochiensis* [14] genome sequences suggests that there is limited overlap between cyst and root-knot nematode effector repertoires. Some of the other sequences on the effector list are consistent with a role in parasitism and include cell wall degrading enzymes, proteinases and venom allergen proteins. In addition, several different transthyretin-like sequences are present. Similar sequences are present in many nematodes, often as large gene families of secreted proteins; *C. elegans* contains more than 60 such sequences. Although a small number of transthyretin-like proteins have been identified as being expressed in the gland cells of several different PPN (reviewed in [4]) their functions remain unknown.

## Conclusions

We have identified a new DNA motif present in the promoter region of the pine wood nematode *B. xylophilus* which is associated with expression in parasitism-specialized tissues – the pharyngeal gland cells. We have validated this promoter motif by in situ hybridisation and through analysis of a gland cell transcriptome dataset. The combination of these approaches allows us to predict novel effector genes. The results described in this study represent a unique opportunity to develop a better understanding of the mechanisms by which the insect-vectored migratory plant-parasitic nematode *B. xylophilus* infects its hosts.

## Methods

### In silico identification of DNA motifs in promoter regions

To identify putative promoter motifs, sequences up to 300 bp upstream of the predicted start codon of *B. xylophilus* genes were extracted from the genome assembly (total of 17,735 promoter regions analyzed from the 18,074 predicted in version 1.2 of the genome) using the script get_upstream_regions.py (https://github.com/peterthorpe5/public_scripts/tree/master/genomic_upstream_regions). To identify potential motifs associated with effectors a list (Additional file 1: Table S1) of verified effector promoters was compared to a similarly sized list of non-effector promoters using the differential motif discovery algorithm HOMER [27]. Occurrences of specific motifs were identified using the FIMO webserver (version 4.11). To determine whether motif occurrences were non-random and not a function of base composition, promoter regions of interest were randomized and the number of motif occurrences in these shuffled promoter regions was counted using custom python script Shuffle_promoter_and_count_occurances_of_motif_per_seq_with_counter_display.py (https://github.com/sebastianevda/SEvdA_promoter_regexp_and_shuffle). The presence of a signal peptide in the associated genes was analysed using SignalP version 4.1 [28]. The bioinformatic pipeline is described in Additional file 6: Table S6.

### In situ hybridisation

The spatial expression patterns of selected genes associated with the predicted motif and/or that were present in the gland cell transcriptome dataset (below) was determined by in situ hybridisation as previously described [9, 29]. The primers used for this analysis are shown in Additional file 7: Table S7.

### Microaspiration of pharyngeal gland cells from *B. xylophilus*

A Portuguese isolate of *B. xylophilus* was cultured on *Botrytis cinerea* in flasks for 7 days at 25 °C [30]. Mixed life stages of the nematodes were collected using the Baermann funnel technique [29] and washed in PBS buffer. Live nematodes were cut using a vibrating razor blade in PBS buffer supplemented with SUPERase-In RNase inhibitor (Life Technologies) to release intact gland cells and fixed in 100% ethanol at − 80 °C overnight. Fixed, cut nematodes were stained in Histogene staining solution (for nucleic acids) (Thermo Fisher Scientific) and resuspended in Halocarbon oil 700 (Sigma). The stained tissues were spread on RNAse free glass cover slips and stored at − 80 °C before further processing. Microaspiration of the pharyngeal gland cells was performed under vacuum on an inverted microscope as previously described and extracted gland cells were stored under oil at − 80 °C before RNA extraction [12].

### RNA sequencing

Total RNA was isolated from approximately 200 mixed dorsal and subventral gland cells using the Arcturus PicoPure RNA isolation kit (Thermofisher Scientific) following the manufacturer’s instructions. Approximately 4 ng of total RNA was isolated from these gland cells following this process. Using the SMARTer Stranded Total RNA-Seq kit - Pico Input Mammalian (Clontech, USA), the total RNA was amplified, ribosomal cDNA was depleted and after a final PCR amplification libraries were sequenced. The quality of the RNA and cDNA was assessed using a Bioanalyzer. Two paired-end libraries (BX-1 and BX-2) were sequenced using the Illumina NextSeq service from Admera Health (USA). These two technical replicates represent one biological replicate (gland cells from mixed life-stage nematodes). The run was spiked with 15–20% PhiX. Raw sequence reads are available under ENA accession number PRJEB24347.

### Analysis of gland cell transcriptome

The RNAseq data from the two libraries generated approximately 268 million paired end reads per library. The reads were trimmed of adapter sequences and low quality bases (Phred < 25) using Trimmomatic v0.32 [31] and aligned to the *B. xylophilus* genome using Tophat2 [32]. Version 1.2 of the genome was used for this analysis and is available at Gene DB ([16], http://www.genedb.org/Homepage/Bxylophilus). The number of reads aligned to each gene were counted using Bedtools, and TMM normalized using Trinity wrapper scripts [33]. *B. xylophilus* genes were sorted into bins of ascending numbers of reads mapped (< 2, 2.1–10, 10.1–100, 100.1–1000, 1000.0–10,000 and > 20,000 FPKM, Fragments per kilobase per million). The proportion of sequences in each bin with a signal peptide (identified using SignalP v4.1) was compared to the proportion of such proteins in the whole genome [34]. The analysis of gene/protein function was based on sequence similarity and performed against non-redundant database by BlastP and Blastn (*e-value* < 1e-04) (NCBI non-redundant protein database (NR), circa January 2017), using a local installation of the Galaxy platform [35]. The bioinformatic pipeline used is described in Additional file 5: Table S5.

## Additional files

Additional file 1: **Table S1.** List of verified effector genes from *Bursaphelenchus xylophilus* used for the analysis of the promoter regions using the differential motif discovery algorithm HOMER. The venny diagram compares the genes in the input effector list with the results from the first output from FIMO analysis. The motif STATWWAWRS is present in the promoter region of 26 genes verified effector genes, which represents approximatly 62% of the genes. (XLSX 102 kb)

Additional file 2: **Table S2.** Nematode genes that have at least one occurence (repetition) of the motif STATAWAARS in the promoter region. The predicted presence or absence of the signal peptide is indicated by 1 or 0 (zero), respectively. (XLSX 48 kb)

Additional file 3: **Table S3.** RNAseq mapped data from the two samples sequenced (BX-1, BX-2). (PDF 22 kb)

Additional file 4: **Table S4.** Top 30 most highly represented genes in the gland cells tissues. SP: presence or absence of signal peptide; motif: presence of at least one repetition of the STATAWAARS motif; ISH: validated the spatial expression; NA: no signal; GC: signal in gland cells. (PDF 35 kb)

Additional file 5: **Table S5.** Limited overlap (venny diagram) between the top most abundant thirty genes represented in the gland cells and the list of 42 genes used for the discovery of DNA sequence motif. (XLSX 68 kb)

Additional file 6: **Table S6.** Bioinformatic pipeline used for the in silico identification of DNA motifs in the promoter regions and the analysis of the *B. xylophilus* pharyngeal gland cells transcriptome. (DOCX 128 kb)

Additional file 7: **Table S7.** List of ten genes that have the presence of STATAWAARS motif and where selected to examine the spatial expression pattern in the nematode tissues (by in situ hybridisation). For each selected gene: Sequence similarity analysis with BlastP, primers used for in situ hybridisation and the results of the validation. (PDF 26 kb)

## Abbreviations

bp: Base pair
BUX: *Bursaphelenchus xylophilus*
DE: Differentially expressed
DOGbox: Dorsal gland cell motif
EST: Expressed Sequence Tag
FPKM: Fragments per kilobase milion
GC: Pharyngeal gland cells
ISH: In situ hybridisation
NR: Non-redundant database
PPN: Plant-parasitic nematodes
RNAseq: Sequencing of RNA
SP: Signal peptide
