# Correction to: STATAWAARS: a promoter motif associated with spatial expression in the major effector-producing tissues of the plantparasitic nematode *Bursaphelenchus xylophilus*

**DOI:** 10.1186/s12864-018-4990-5

**Published:** 2018-08-10

**Authors:** Margarida Espada, Sebastian Eves-van den Akker, Tom Maier, Paramasivan Vijayapalani, Thomas Baum, Manuel Mota, John T. Jones

**Affiliations:** 1 NemaLab, ICAAM – Instituto de Ciências Agrárias e Ambientais Mediterrânicas, Universidade de Évora, Instituto de Investigação e Formação Avançada, Universidade de Évora, Pólo da Mitra, Ap. 94, 7006-554 Évora, Portugal; 2 Cell and Molecular Sciences Group, The James Hutton Institute, Invergowrie, Dundee, DD2 5DA UK; 3 Department of Plant Sciences, University of Cambridge, Cambridge, CB3 1AB UK; 4 Department of Plant Pathology and Microbiology, Iowa State University, Ames, 50011 USA; 5 NemaLab, ICAAM - Departamento de Biologia, Instituto de Ciências Agrárias e Ambientais Mediterrânicas, Universidade de Évora, Escola de Ciências e Tecnologia, Universidade de Évora, Pólo da Mitra, Ap. 94, 7006-554 Évora, Portugal; 6 School of Biology, University of St Andrews, North Haugh, St Andrews, KY16 9TZ UK

## Correction

Following publication of the original article [1], the authors reported that one of the authors’ names was erroneously changed during proofing and published incorrectly. In this Correction the incorrect and correct author name are shown. The original publication of this article has been corrected.

Originally the author name has been published as:

- Vijayapalani Paramasivan

The correct author name is:

- Paramasivan Vijayapalani
