# Supplementary material for: STATAWAARS: a promoter motif associated with spatial expression in the major effector-producing tissues of the plant-parasitic nematode Bursaphelenchus xylophilus
Source: BMC Genomics. 2018 Jul 27;19:553. doi: 10.1186/s12864-018-4908-2 (PMC6062891; doi:10.1186/s12864-018-4908-2)
Supplement: Supplementary file 3 — Table S3. RNAseq mapped data from the two samples sequenced (BX-1, BX-2). (PDF 22 kb) [file 12864_2018_4908_MOESM3_ESM.pdf]

Table S3– RNAseq mapped data from the two samples sequenced (BX-1, BX-2).

	**Trimmed reads input**	**Mapped reads**
Bx-1	124218810	37820234 (*30.4% of input*)
Bx-2	143259452	34916231 (*24.4% of input*)
