# Supplementary material for: STATAWAARS: a promoter motif associated with spatial expression in the major effector-producing tissues of the plant-parasitic nematode Bursaphelenchus xylophilus
Source: BMC Genomics. 2018 Jul 27;19:553. doi: 10.1186/s12864-018-4908-2 (PMC6062891; doi:10.1186/s12864-018-4908-2)
Supplement: Supplementary file 4 — Table S4. Top 30 most highly represented genes in the gland cells tissues. SP: presence or absence of signal peptide; motif: presence of at least one repetition of the STATAWAARS motif; ISH: validated the spatial expression; NA: no signal; GC: signal in gland cells. (PDF 35 kb) [file 12864_2018_4908_MOESM4_ESM.pdf]

Table S4 - Top 30 most highly represented genes in the gland cells tissues. SP: presence or absence of signal peptide; motif: presence of at least one repetition of the STATAWAARS motif; ISH: validated the spatial expression; NA: no signal; GC: signal in gland cells.

(1) According to *Bursaphelenchus xylophilus* genome version 1.2 (available in Gene DB)

(2) *in situ* hybridization

(3) based on Shinya et al., 2013

(4) based on results from Espada *et al.*, 2016, *Mol. Plant Pathol.*

Gene ID[1]	expression (FPKM)	SP	Motif	ISH[2]	Secretome[3]	post infection[4]	Gene Description (based on sequence similarity)
BUX.s01226.3	43610,67	+	+			-	Reverse transcriptase
BUX.s01226.2	34330,2	+	-			DPI	Reverse transcriptase
BUX.s00083.32	28320,67	+	-		+	FF	Cysteine peptidase, family C1A (Proteinase inhibitor I29)
BUX.s01513.259	28133,97	-	-			DPI	Not known
BUX.s00364.143	20947,55	-	-			DPI	Hypothetical protein; Metridin-like ShK toxin
BUX.s01281.223	20907,19	+	+	GC		DPI	Not known
BUX.s00782.2	16572,97	-	+			FF	Small HSP21-like protein; HSP20-like
BUX.s01063.193	14530,48	+	+			-	Transthyretin-like family protein
BUX.s01109.570	13699,99	+	-	NA		DPI	Not known
BUX.s01332.1	12613,75	+	-			DPI	Not known
BUX.s01639.10	12613,75	+	-			-	Not known
BUX.s01167.27	9072,31	-	-			DPI	Not known
BUX.s01144.234	8343,09	+	+	GC	+	DPI	Thaumatin-like protein 1b
BUX.s01144.128	8146,98	+	-		+	DPI	Not known
BUX.s00532.10	7085,05	+	+	NA		DPI	Aspartic Peptidase, family A1
BUX.s00036.112	6830,64	+	+	GC	+	DPI	Beta-1,4-endoglucanase; GH45
BUX.s01259.45	6760,58	+	+	NA	+	DPI	Cysteine protease family cathepsin 1; Proteinase inhibitor I29
BUX.s01143.167	6745,18	-	-			DPI	Not known
BUX.s00647.61	5542,85	+	+		+	DPI	Not known
BUX.s01167.26	5473,3	-	-			DPI	
BUX.s01109.169	5433,02	+	-			-	Saposin B domain
BUX.s01226.4	5245	-	-			DPI	Hypothetical protein - common roundworm retrotransposon R4
BUX.s01226.1	5037,37	-	-			DPI	Hypothetical protein - common roundworm retrotransposon R4
BUX.s00036.113	4504,83	+	+	GC	+	DPI	Beta-1,4-endoglucanase (*B. xylophilus*)
BUX.s00298.157	4309,6	-	-			DPI	Eukaryotic translation elongation factor 1A protein (*B. xylophilus*)
BUX.s01144.122	4148,47	+	+	GC		DPI	Not known
BUX.s00713.953	3997,31	+	+	NA	+	DPI	Peptidase aspartic, family A1
BUX.s00139.22	3881,41	+	+		+	-	Not known
BUX.s01147.119	3703,45	-	-			-	Calreticulin (*B. xylophilus*)
BUX.s01063.196	3408,99	+	-			DPI	Not known
