# Supplementary material for: STATAWAARS: a promoter motif associated with spatial expression in the major effector-producing tissues of the plant-parasitic nematode Bursaphelenchus xylophilus
Source: BMC Genomics. 2018 Jul 27;19:553. doi: 10.1186/s12864-018-4908-2 (PMC6062891; doi:10.1186/s12864-018-4908-2)
Supplement: Supplementary file 6 — Table S6. Bioinformatic pipeline used for the in silico identification of DNA motifs in the promoter regions and the analysis of the B. xylophilus pharyngeal gland cells transcriptome. (DOCX 128 kb) [file 12864_2018_4908_MOESM6_ESM.docx]

**Table S6 –** Bioinformatic pipeline used for the *in silico* identification of DNA motifs in the promoter regions and the analysis of the *B. xylophilus* pharyngeal gland cells transcriptome.

***In silico* identification of DNA motifs in promoter regions**

python /HOME/get_upstream_regions_using_gene_coordinates_GFF_format_plus_gene_region.py -c BUX.v1.2.all.mod.gff3_for_py_script.out -g BurXv1.2.supercontigs.fa -f all_BUX.v1.2_gene_names.out -u 300 -o BUXv1.2_all_gene_names.out.300up.fasta > warning_BUXv1.2_all_gene_names.out.300up.out

findMotifs.pl /home/…/genomic_upstream_regions/BUXv1.2_42_gene_names.out.300up.fasta fasta /home/me42047/scripts/genomic_upstream_regions/42_vs_all/ -fasta /home/me42047/scripts/genomic_upstream_regions/BUXv1.2_all_gene_names.out.300up.fasta

**Analysis of the gland cell transcriptome**

java -jar /home/me42047/downloads/Trimmomatic-0.32/Trimmomatic-0.32/trimmomatic-0.32.jar PE -threads 16 -trimlog /home/me42047/Bursaphelenchus_xylophilus/working_data/2017_glandcell_seq/trimmed_reads/trimlog.txt /home/me42047/Bursaphelenchus_xylophilus/working_data/2017_glandcell_seq/raw_data/1703-TM103_S0_L003_R1_001.fastq /home/me42047/Bursaphelenchus_xylophilus/working_data/2017_glandcell_seq/raw_data/1703-TM103_S0_L003_R2_001.fastq /home/me42047/Bursaphelenchus_xylophilus/working_data/2017_glandcell_seq/trimmed_reads/out_pair_1703-TM103_S0_L003_R1_001.fastq /home/me42047/Bursaphelenchus_xylophilus/working_data/2017_glandcell_seq/trimmed_reads/out_un_pair_1703-TM103_S0_L003_R1_001.fastq /home/me42047/Bursaphelenchus_xylophilus/working_data/2017_glandcell_seq/trimmed_reads/out_pair_1703-TM103_S0_L003_R2_001.fastq /home/me42047/Bursaphelenchus_xylophilus/working_data/2017_glandcell_seq/trimmed_reads/out_un_pair_1703-TM103_S0_L003_R2_001.fastq LEADING:22 TRAILING:22 SLIDINGWINDOW:10:22 MINLEN:30

java -jar /home/me42047/downloads/Trimmomatic-0.32/Trimmomatic-0.32/trimmomatic-0.32.jar PE -threads 16 -trimlog /home/me42047/Bursaphelenchus_xylophilus/working_data/2017_glandcell_seq/trimmed_reads/trimlog.txt /home/me42047/Bursaphelenchus_xylophilus/working_data/2017_glandcell_seq/raw_data/1703-TM104_S0_L003_R1_001.fastq /home/me42047/Bursaphelenchus_xylophilus/working_data/2017_glandcell_seq/raw_data/1703-TM104_S0_L003_R2_001.fastq /home/me42047/Bursaphelenchus_xylophilus/working_data/2017_glandcell_seq/trimmed_reads/out_pair_1703-TM104_S0_L003_R1_001.fastq /home/me42047/Bursaphelenchus_xylophilus/working_data/2017_glandcell_seq/trimmed_reads/out_un_pair_1703-TM104_S0_L003_R1_001.fastq /home/me42047/Bursaphelenchus_xylophilus/working_data/2017_glandcell_seq/trimmed_reads/out_pair_1703-TM104_S0_L003_R2_001.fastq /home/me42047/Bursaphelenchus_xylophilus/working_data/2017_glandcell_seq/trimmed_reads/out_un_pair_1703-TM104_S0_L003_R2_001.fastq LEADING:22 TRAILING:22 SLIDINGWINDOW:10:22 MINLEN:30

/home/me42047/downloads/FastQC/fastqc /home/me42047/Bursaphelenchus_xylophilus/working_data/2017_glandcell_seq/trimmed_reads/out_pair_1703-TM103_S0_L003_R1_001.fastq /home/me42047/Bursaphelenchus_xylophilus/working_data/2017_glandcell_seq/trimmed_reads/out_pair_1703-TM103_S0_L003_R2_001.fastq/ /home/me42047/Bursaphelenchus_xylophilus/working_data/2017_glandcell_seq/trimmed_reads/out_pair_1703-TM104_S0_L003_R1_001.fastq /home/me42047/Bursaphelenchus_xylophilus/working_data/2017_glandcell_seq/trimmed_reads/out_pair_1703-TM104_S0_L003_R2_001.fastq -t 16

mapping pair_reads with Tophat2.1.1 for sample 103 (output dir tophatout_103)

/home/me42047/downloads/tophat-2.1.1.Linux_x86_64/tophat2 -o /home/me42047/Bursaphelenchus_xylophilus/working_data/2017_glandcell_seq/tophatout_103 -r 350 -i 50 --solexa-quals -p 8 --no-discordant /home/me42047/Bursaphelenchus_xylophilus/working_data/bowtie2_indexes/BurXv1.2.supercontigs /home/me42047/Bursaphelenchus_xylophilus/working_data/2017_glandcell_seq/trimmed_reads/out_pair_1703-TM103_S0_L003_R1_001.fastq,/home/me42047/Bursaphelenchus_xylophilus/working_data/2017_glandcell_seq/trimmed_reads/out_pair_1703-TM103_S0_L003_R2_001.fastq

/home/me42047/downloads/tophat-2.1.1.Linux_x86_64/tophat2 -o /home/me42047/Bursaphelenchus_xylophilus/working_data/2017_glandcell_seq/tophatout_103 -r 350 -i 50 --solexa-quals -p 8 --no-discordant /home/me42047/Bursaphelenchus_xylophilus/working_data/bowtie2_indexes/BurXv1.2.supercontigs /home/me42047/Bursaphelenchus_xylophilus/working_data/2017_glandcell_seq/trimmed_reads/out_pair_1703-TM103_S0_L003_R1_001.fastq,/home/me42047/Bursaphelenchus_xylophilus/working_data/2017_glandcell_seq/trimmed_reads/out_pair_1703-TM103_S0_L003_R2_001.fastq

samtools sort -o /home/me42047/Bursaphelenchus_xylophilus/working_data/2017_glandcell_seq/gene_counts/out.103accepted_hits.bam /home/me42047/Bursaphelenchus_xylophilus/working_data/2017_glandcell_seq/gene_counts/103accepted_hits.bam

samtools sort -o /home/me42047/Bursaphelenchus_xylophilus/working_data/2017_glandcell_seq/gene_counts/out.104accepted_hits.bam /home/me42047/Bursaphelenchus_xylophilus/working_data/2017_glandcell_seq/gene_counts/104accepted_hits.bam

samtools index -b out.103accepted_hits.bam

samtools index -b out.104accepted_hits.bam

grep 'gene' BUX.v1.2.phase.correct.gff3 > BUXv1.2.all.mod.gff3

bedtools multicov -bams out.103accepted_hits.bam out.104accepted_hits.bam -bed BUXv1.2.all.mod.gff3 > out_GC_BUX_1.2_gene_counts.gff

awk '{print $3, $9, $10, $12, $13, $14, $15, $16, $17, $18, $19, $20, $21, $22, $23}' out_GC_BUX_1.2_gene_counts.gff > sorted.out_GC_BUX_1.2_gene_counts.gff

awk {'print $1, $2, $4, $5}' sorted.out_GC_BUX_1.2_genecounts > final_sorted.out_GC_BUX_1.2_genecounts

/home/me42047/downloads/trinityrnaseq-Trinity-v2.3.2/Analysis/DifferentialExpression/run_TMM_normalization_write_FPKM_matrix.pl --matrix final_sorted.out_GC_BUX_1.2_genecounts --lengths feature_length_v1_2.txt
