# Supplementary material for: STATAWAARS: a promoter motif associated with spatial expression in the major effector-producing tissues of the plant-parasitic nematode Bursaphelenchus xylophilus
Source: BMC Genomics. 2018 Jul 27;19:553. doi: 10.1186/s12864-018-4908-2 (PMC6062891; doi:10.1186/s12864-018-4908-2)
Supplement: Supplementary file 7 — Table S7. List of ten genes that have the presence of STATAWAARS motif and where selected to examine the spatial expression pattern in the nematode tissues (by in situ hybridisation). For each selected gene: Sequence similarity analysis with BlastP, primers used for in situ hybridisation and the results of the validation. (PDF 26 kb) [file 12864_2018_4908_MOESM7_ESM.pdf]

Table S7 – List of ten genes that have the presence of STATAWAARS motif and where selected to examine the spatial expression pattern in the nematode tissues (by *in situ* hybridization). For each selected gene: Sequence similarity analysis with BlastP, primers used for *in situ* hybridization and the results of the validation.

Gene ID	Forward primer	Reverse primer	Sequence similarity	Localisation in the nematode tissues
**BUX.s01144.234**	TGTCAAGATCACGGTCGTCA	TTCCACAAGCACCAGTTTCG	Thaumatin-like protein	Gland cells
**BUX.s01109.106**	TATGACGTGGACACCCTCAG	GCGGCCTTGTCAGATTCTTT	Pioneer	Gland cells
**BUX.s01145.19**	ACAGCTGCCCCAATGATTAC	CCGCATTGATTACGTTGATG	Alpha-beta hydrolase	Gland cells
**BUX.s01147.71**	CAAGGAGTAGCGGTGAGAGG	TTCTCAGTTCGGGTTCGATT	Transthyretin-like protein	Gland cells
**BUX.s01066.8**	AAACCGCAACGTGAAAGTGA	GACACCGTATTGTTTGGCGA	Lysozyme	Instestine
**BUX.s00713.837**	TCTTCCATTTGAAGCCATCC	GTGGACTCAAAGCAACGTCA	Pioneer	Intestine
**BUX.s00060.1**	ACTTCACTCTGCTGCCTTCT	GACCGTGTCCAAGTTCCTAA	Pioneer	No signal
**BUX.s00647.61**	GCCGACCTTGTTGAACTTGT	TGTCCGACCTTCTGCAAGAT	Pioneer	Gland cells
**BUX.s00713.1002**	CCATGCTGTACGAGCTGAAA	AATCGGAAAGTCGAACATGG	Alpha-beta hydrolase	Gland cells
**BUX.s00116.969**	TGGACGTCCTCATCATCAGA	GATTTGGGGTGAAGGGTCTT	Pioneer	Gland cells
